# Breast Augmentation Patient Satisfaction in an Appalachian Region

**DOI:** 10.7759/cureus.62550

**Published:** 2024-06-17

**Authors:** Armein Rahimpour, Samuel Suite, Mathew Dudich, David A Denning, Paul Bown, Peter Ray, Rahman Barry

**Affiliations:** 1 General Surgery, Marshall University Joan C. Edwards School of Medicine, Huntington, USA; 2 Plastic Surgery, UK King's Daughters Medical Center, Ashland, USA; 3 Plastic and Reconstructive Surgery, Marshall University Joan C. Edwards School of Medicine, Huntington, USA

**Keywords:** patient satisfaction score, rural appalachia, breast augmentation, rural healthcare disparities, breast implant

## Abstract

The prevalence of cosmetic plastic surgeries, including breast augmentation, has risen significantly, with breast augmentation being among the most sought-after procedures. However, there's a dearth of research on patient outcomes and satisfaction, particularly in rural areas like the Appalachian region. This retrospective study aimed to fill this gap by examining patient satisfaction and complications following breast augmentation surgery among rural Appalachian patients in the tri-state (West Virginia, Kentucky, and Ohio) area. A total of 63 patients who underwent primary breast augmentation at a regional referral center from June 2014 to December 2022 were included in the study. Patient records were reviewed and data on demographics, complications, re-operations, and satisfaction scores were analyzed. Results revealed no significant differences between rural and urban populations in terms of demographic characteristics, complication rates, re-operation rates, or satisfaction scores. Logistic regression models confirmed that rural/urban status did not significantly influence the likelihood of complications, re-operations, or satisfaction.

Despite the study's limitations, including a small sample size and single-center design, the results indicate that rural Appalachian patients receive surgical care comparable to their urban counterparts and experience similar benefits from breast augmentation surgery. Recognizing the distinctive healthcare needs and obstacles faced by rural communities is essential for mitigating healthcare disparities and enhancing overall health outcomes. Future research and healthcare initiatives should prioritize improving access to care, fostering patient-centered approaches, and addressing systemic challenges in healthcare delivery across rural Appalachia.

## Introduction

The prevalence of cosmetic plastic surgeries, including breast augmentation, has surged in the United States over the years, reflecting a growing trend toward enhancing physical appearance and bolstering self-esteem. In 2022 alone, just under 1.5 million cosmetic surgical procedures were performed, with breast augmentation ranking among the top three most sought-after procedures [1]. Studies have extensively documented the multifaceted benefits of breast augmentation, ranging from improvements in overall quality of life to enhanced self-assurance, interpersonal relationships, and sexual well-being [2,3].

Despite the documented benefits, breast augmentation, like any surgical procedure, carries inherent risks and potential complications. These can range from early post-operative issues such as hematoma and infection to late complications like capsular contracture and implant rupture [4]. Given the rising demand for breast augmentation and its associated risks, there has been a paradigm shift in plastic surgery research towards understanding patient perceptions, satisfaction, and quality of life outcomes following surgery [5].

The development of patient-reported outcome measures, such as the BREAST-Q, has revolutionized the assessment of surgical outcomes by capturing patients' perspectives on their esthetic results and psychosocial well-being [6]. The BREAST-Q, introduced in 2009, has become the gold standard for evaluating patient satisfaction and quality of life outcomes following breast surgery, offering insights into various domains, including satisfaction with breasts, overall outcomes, and psychosocial and sexual well-being [6].

While numerous studies have investigated patient satisfaction and outcomes following breast augmentation surgery, there remains a paucity of research focusing on specific geographical regions, particularly rural areas. Rural populations, such as those in the Appalachian region, face unique healthcare challenges characterized by limited access to quality care, socioeconomic disparities, and higher rates of certain health conditions [7,8]. Despite these challenges, there is a dearth of research examining the experiences and outcomes of breast augmentation surgery among rural Appalachian populations.

Understanding the unique healthcare needs and experiences of rural Appalachian patients undergoing breast augmentation is paramount for addressing healthcare disparities and improving overall well-being. However, to date, no studies have investigated breast augmentation satisfaction specifically from the standpoint of Appalachian patients, particularly within the tri-state (West Virginia, Kentucky, and Ohio) area.

Therefore, this study aimed to fill this critical gap in the literature by identifying patient satisfaction and complications of breast augmentation surgery among Appalachian patients, with a specific focus on the tri-state area. By elucidating the experiences and outcomes of breast augmentation surgery in this population, this research seeks to inform healthcare providers and policymakers on ways to improve access to care and enhance patient outcomes in rural Appalachian communities.

## Materials and methods

The study was authorized by the Institutional Review Board of Marshall University (IRB no. 2110186-2). In order to complete this retrospective study, patient records maintained in Cabell Huntington Hospital's electronic medical record database were reviewed. Cabell Huntington Hospital (CHH) is an American College of Surgeons-verified Level II Trauma Center in Huntington, WV, that serves as a regional referral center for patients of the tri-state (West Virginia-Kentucky-Ohio) area.

The medical records reviewed pertained to patients who underwent primary esthetic breast augmentation at CHH from June 2014 to December 2022, spanning 7.5 years. The CHH Information Technology (IT) department was engaged to retrieve the data, encompassing patients undergoing primary breast augmentation for esthetic purposes. We specifically requested patient identifiers such as age, gender, race, medical record number (MRN), date of birth, and zip code from the IT team. Initially, our sample comprised 87 patients. Subsequently, all collected data were centralized using Microsoft Excel software (Redmond, WA: Microsoft Corp.). After data collection, we extracted additional information from the medical records, including contact details, original operation dates, capsular contraction, asymmetry, malposition, hematoma formation, and instances requiring revision or a return to the operating room. Following chart review, our final sample size was 63, excluding patients with a history of cancer or those under the age of 18 years.

To classify the population into rural and urban categories, we utilized zip codes that were provided from chart review at the time of surgery. Our study was grounded on the BREAST-Q Augmentation Module, serving as the foundation for our research. Each of the module's 15 questions was rated on a scale from 1 to 4, with higher scores indicating greater satisfaction. Aggregating the scores from these questions resulted in a scale ranging from 15 to 64. Using contact information obtained from electronic medical records, we reached out to patients and invited them to participate in the survey. We ensured full disclosure regarding the time commitment for participation and guaranteed anonymity. Patients were contacted no more than three times using hospital phone numbers. Ultimately, 12 patients who had undergone breast augmentation at CHH agreed to participate in the study. During the survey, patients were verbally presented with the questions from the BREAST-Q Augmentation Module, and their responses were electronically recorded (Table 1).

**Table 1:** Breast-Q survey questionnaire.

Questions	Very dissatisfied	Somewhat dissatisfied	Somewhat satisfied	Very satisfied
How do you look in the mirror clothed?	1	2	3	4
The shape of our reconstructed breast(s) when you are wearing a bra?	1	2	3	4
How normal do you feel in your clothes?	1	2	3	4
The size of your reconstructed breast(s)?	1	2	3	4
Being able to wear clothing that is more fitted?	1	2	3	4
How are your breast area lined up in relation to each other?	1	2	3	4
How comfortably do your bras fit?	1	2	3	4
The softness of our reconstructed breast(s)?	1	2	3	4
How equal in size your breasts are to each other?	1	2	3	4
How naturally your reconstructed breast(s) sits/hangs?	1	2	3	4
How your reconstructed breast(s) feels to touch?	1	2	3	4
How much your reconstructed breast(s) feels like a natural part of your body?	1	2	3	4
How closely matched your breasts are to each other?	1	2	3	4
How do you look in the mirror unclothed?	1	2	3	4

Descriptive statistics, including means, standard deviations (SDs), frequencies, and percentages, were computed to summarize participant demographics and outcome variables. To examine differences between rural and urban populations, Student’s t-test tests were utilized for continuous variables such as age, BMI, and satisfaction scores. Chi-squared tests were applied to categorical variables, including race, diabetes mellitus, tobacco use, capsular contraction, asymmetry, hematoma, re-operations, and satisfaction categories. Logistic regression was utilized to assess the association between rural and urban populations and three categorical outcomes as follows: complications, re-operations, and satisfaction. For odds ratios, 95% confidence intervals (CIs) were calculated. All statistical analyses were conducted using SAS version 9.4 (Cary, NC: SAS Institute Inc.). Statistical significance was set at p<0.05 for all analyses.

## Results

Comparison of rural and urban population characteristics

Descriptive statistics comparing rural and urban areas are presented in Table 2. The study encompassed 37 participants from rural areas and 26 participants from urban areas. Table 1 illustrates that there were no statistically significant differences between rural and urban populations concerning age (mean rural=41, SD rural=12.0; mean urban=45, SD urban=14; p=0.12) or body mass index (BMI) (mean rural=26.0, SD rural=5.5; mean urban=25.6, SD urban=5.0, p=0.94).

**Table 2:** Overall patient characteristics and complications categorized based on urban and rural area status. Data presented as n (%), excluding SD. The notation "missing n" indicates that the variable was not identified during the chart review process. This means that specific information for certain patients, such as demographics or clinical characteristics, was either not recorded or unavailable in the data collected. n: number; BMI: body mass index; SD: standard deviation

Variables	Overall (n=63)	Rural (n=37)	Urban (n=26)	p-Value
Age				
Mean±SD	43±13	41±12	45±14	0.12
Race				
White	60 (95)	37 (100)	23 (88)	0.065
Hispanic	1 (1.6)	0 (0)	1 (3.8)	
Multiple	2 (3.2)	0 (0)	2 (7.7)	
Diabetes mellitus				
No	61 (97)	35 (95)	26 (100)	0.51
Yes	2 (3.2)	2 (5.4)	0 (0)	
Tobacco				
No	49 (78)	28 (76)	21 (81)	0.63
Yes	14 (22)	9 (24)	5 (19)	
BMI				
Mean±SD	25.9±5.3	26.0±5.5	25.6±5.0	0.94
Missing n	14	6	8	
Complication				
No	49 (78)	29 (78)	20 (77)	0.89
Yes	14 (22)	8 (22)	6 (23)	
Capsular contraction				
No	58 (92)	34 (92)	24 (92)	0.99
Yes	5 (7.9)	3 (8.1)	2 (7.7)	
Asymmetry				
No	60 (95)	36 (97)	24 (92)	0.56
Yes	3 (4.8)	1 (2.7)	2 (7.7)	
Malposition				
No	63 (100)	37 (100)	26 (100)	NA
Hematoma				
No	62 (98)	36 (97)	26 (100)	0.99
Yes	1 (1.6)	1 (2.7)	0 (0)	
Re-operation				
No	51 (81)	30 (81)	21 (81)	0.99
Yes	12 (19)	7 (19)	5 (19)	

In terms of demographic characteristics, no significant differences were observed in racial composition between rural (100% White) and urban (88% White) populations (p=0.065). Likewise, the prevalence of diabetes and tobacco use did not significantly differ between rural and urban populations (diabetes: p=0.51; tobacco: p=0.63). Regarding medical outcomes, no significant differences were found in complication rates and re-operations between rural and urban populations (complication: p=0.89; re-operations: p=0.99).

Table 3 presents the results of outcome comparisons using logistic regression models. For complications, the odds ratio was 0.92 (95% CI: 0.28, 3.18), with a p-value of 0.90, indicating no significant association between rural/urban status and the likelihood of experiencing complications. Similarly, for re-operation, the odds ratio was 0.98 (95% CI: 0.27, 3.71), with a p-value of 0.92, suggesting no significant difference in the odds of requiring re-operation between rural and urban populations.

**Table 3:** Comparing outcomes using logistic and linear regression models. The urban population was used as the reference group.

Outcomes	Odds ratio or beta estimate	95% CI	p-Value
Complication	0.92	0.28, 3.18	0.90
Re-operation	0.98	0.27, 3.71	0.92

Participant characteristics and survey responses

All patients were contacted, but only 12 participated in the survey. Descriptive statistics comparing rural and urban areas for survey participants are presented in Table 4. The completed survey group comprised eight participants from rural areas and four from urban areas. Table 4 demonstrates no statistically significant differences between rural and urban populations in terms of age (mean rural=46, SD rural=14.0; mean urban=46, SD urban=9; p=0.86), body mass index (BMI) (mean rural=27.9, SD rural=6.7; mean urban=24.5, SD urban=4.0; p=0.32), and satisfaction scores (mean rural=50.0, SD rural=11.0; mean urban=57.0, SD urban=4.0; p=0.35). Furthermore, no significant differences were found in racial composition between rural (100% White) and urban (100% White) populations. The prevalence of diabetes and tobacco use also did not significantly differ between rural and urban populations. In terms of medical outcomes, no significant differences were found in complication rates and re-operations between rural and urban populations (complication: p=0.98; re-operations: p=0.49).

**Table 4:** Patient characteristics, complications, and satisfaction scores were categorized based on urban and rural area status for participators in the BREAST-Q survey. Data presented as n (%), excluding SD. The notation "missing N" indicates that the variable was not identified during the chart review process. This means that specific information for certain patients, such as demographics or clinical characteristics, was either not recorded or unavailable in the data collected. n: number; BMI: body mass index; SD: standard deviation

Variables	Overall (n=12)	Rural (n=8)	Urban (n=4)	p-Value
Age				
Mean±SD	46±12	46±14	46±9	0.86
Race				
White	12 (100)	8 (100)	4 (100)	NA
Diabetes mellitus				
No	12 (100)	8 (100)	4 (100)	NA
Tobacco				
No	10 (83)	7 (88)	3 (75)	0.97
Yes	2 (17)	1 (13)	1 (25)	
BMI				
Mean±SD	26.7±5.9	27.9±6.7	24.5±4.0	0.32
Missing N	1	1	0	
Complication				
No	8 (67)	5 (63)	3 (75)	0.98
Yes	4 (33)	3 (38)	1 (25)	
Capsular contraction				
No	9 (75)	6 (75)	3 (75)	0.99
Yes	3 (25)	2 (25)	1 (25)	
Asymmetry				
No	12 (100)	8 (100)	4 (100)	NA
Malposition				
No	12 (100)	8 (100)	4 (100)	NA
Hematoma				
No	11 (92)	7 (88)	4 (100)	0.98
Yes	1 (8.3)	1 (13)	0 (0)	
Re-operation				
No	9 (75)	5 (63)	4 (100)	0.49
Yes	3 (25)	3 (38)	0 (0)	
Patient satisfaction scores				
Mean±SD	52±10	50±11	57±4	0.35
Patient satisfaction				
Dissatisfied	0 (0)	0 (0)	0 (0)	0.66
Somewhat dissatisfied	1 (8.3)	1 (12)	0 (0)	
Somewhat satisfied	2 (17)	2 (25)	0 (0)	
Satisfied	9 (75)	5 (63)	4 (100)	

Table 5 presents the results of outcome comparisons using logistic regression models for survey participants. For complications, the odds ratio was 1.80 (95% CI: 0.14, 46.2), with a p-value of 0.70, indicating no significant association between rural/urban status and the likelihood of experiencing complications. Similarly, for re-operation, the odds ratio was 5.73 (95% CI: 0.23, 142.6), with a p-value of 0.29, suggesting no significant difference in the odds of requiring re-operation between rural and urban populations. Regarding satisfaction scores, the odds ratio was 0.56 (95% CI: 0.018, 16.77), with a p-value of 0.74, indicating no significant association between rural/urban status and the likelihood of satisfaction.

**Table 5:** Comparing outcomes using logistic and linear regression models for patients who participated in the BREAST-Q survey. The urban population was used as the reference group.

Outcomes	Odds ratio or beta estimate	95% CI	p-Value
Complication	1.80	0.14, 46.2	0.70
Re-operation	5.73	0.23, 142.56	0.29
Satisfaction	0.56	0.018, 16.77	0.74

## Discussion

The findings of this study contribute valuable insights into the experiences and outcomes of breast augmentation surgery among rural Appalachian patients within the tri-state (West Virginia, Kentucky, and Ohio) area. Understanding the unique healthcare needs and challenges faced by rural populations is crucial for addressing healthcare disparities and improving overall well-being [6]. This discussion will delve into the implications of the study findings, considering various factors that may influence patient satisfaction, medical outcomes, and access to care in rural Appalachian communities.

Patient demographics and health profiles

Our study revealed no significant differences in demographic characteristics between rural and urban populations undergoing breast augmentation surgery [1]. This suggests that rural Appalachian patients seeking cosmetic surgery share similar demographic profiles with their urban counterparts [6]. The predominantly White racial composition in both rural and urban populations aligns with the demographic makeup of the Appalachian region [7]. However, it's essential to acknowledge the potential influence of cultural and socioeconomic factors on patient decision-making and satisfaction with surgical outcomes [3].

Furthermore, the comparable prevalence of diabetes and tobacco use between rural and urban populations highlights the need for targeted preoperative screening and counseling to mitigate surgical risks [1]. While our study did not find significant disparities in health profiles, future research should explore the impact of socioeconomic status, education level, and healthcare access on patient outcomes in rural Appalachian communities [6].

Methodological rigor

The methodological strengths of this study include the use of validated patient-reported outcome measures such as the BREAST-Q and rigorous statistical analysis techniques to assess the associations between rural/urban status and surgical outcomes [1,9,10]. By incorporating established instruments like the BREAST-Q, we were able to enhance the reliability and validity of our findings, providing a comprehensive assessment of patient satisfaction and quality of life following breast augmentation surgery. However, it's essential to acknowledge the limitations of the study - the small sample size and single-center design. Future research should aim to replicate these findings in larger, multicenter studies with diverse patient populations to enhance the generalizability of the results.

Medical outcomes and complications

Contrary to expectations, our study found no significant differences in complication rates or re-operation rates between rural and urban populations [1]. These findings suggest that rural Appalachian patients have access to quality surgical care and post-operative management comparable to their urban counterparts [7]. However, it's essential to consider potential confounding factors such as surgeon expertise, hospital resources, and patient adherence to postoperative instructions [2].

While our study focused specifically on breast augmentation surgery, the absence of significant disparities in medical outcomes underscores the importance of comprehensive preoperative evaluation and perioperative care in rural healthcare settings [1]. Multidisciplinary collaboration among plastic surgeons, primary care providers, and allied health professionals can optimize patient safety and surgical outcomes in rural communities [6]. Moreover, ongoing monitoring and quality improvement initiatives are essential for identifying and addressing potential barriers to care in remote and underserved areas [7].

Patient satisfaction and quality of life

Despite the lack of significant differences in medical outcomes, our study found no significant disparities in patient satisfaction scores between rural and urban populations [1]. This suggests that rural Appalachian patients derive comparable benefits from breast augmentation surgery in terms of esthetic enhancement and psychosocial well-being [3,8]. The use of validated patient-reported outcome measures, such as the BREAST-Q, enables healthcare providers to assess the holistic impact of surgery on patients' quality of life and satisfaction with outcomes [5]. This aligns with previous research utilizing the BREAST-Q, which has demonstrated its effectiveness in evaluating patient-reported outcomes and esthetic satisfaction [9,11]. The use of validated instruments like the BREAST-Q enables healthcare providers to assess the holistic impact of surgery on patients' well-being, including psychosocial factors and quality of life.

Policy and practice implications

The findings of this study have several implications for healthcare delivery in rural Appalachian communities [1]. Firstly, enhancing access to specialized surgical services and postoperative support services is essential for optimizing patient outcomes and satisfaction [6]. Telehealth initiatives, mobile surgical units, and outreach programs can help bridge the gap in healthcare access for rural residents, particularly those living in remote areas [7].

Secondly, promoting patient-centered care and shared decision-making is critical for ensuring that surgical interventions align with patient preferences, values, and goals [3]. Culturally sensitive communication strategies and educational materials can empower rural Appalachian patients to make informed decisions about cosmetic surgery and actively participate in their care [6].

Thirdly, fostering collaboration among healthcare providers, community organizations, and policymakers is essential for addressing systemic barriers to care and promoting health equity in rural Appalachia [7]. Initiatives aimed at improving healthcare infrastructure, expanding insurance coverage, and reducing socioeconomic disparities can enhance access to comprehensive healthcare services and improve population health outcomes in underserved regions [6].

Future research directions

Future research should continue to explore the utility of patient-reported outcome measures such as the BREAST-Q in evaluating surgical outcomes and patient satisfaction in rural Appalachian communities [9-11]. Longitudinal studies could investigate the long-term effects of breast augmentation surgery on patient well-being, incorporating the perspectives of diverse patient populations and considering socioeconomic factors that may influence outcomes. By expanding our understanding of patient experiences and outcomes, we can further enhance the delivery of cosmetic surgical services and address healthcare disparities in underserved regions [7].

## Conclusions

In conclusion, this study sheds light on the experiences and outcomes of breast augmentation surgery among rural Appalachian patients in the tri-state area, addressing a significant gap in the literature. Despite the unique healthcare challenges faced by rural populations, our findings suggest that patients in these areas receive comparable surgical care and derive similar satisfaction from the procedure as their urban counterparts. The absence of significant disparities in complication rates, re-operation rates, and satisfaction scores underscores the importance of ensuring equitable access to surgical services and postoperative support for rural communities. By recognizing and addressing the healthcare needs of rural populations, healthcare providers and policymakers can take proactive steps to improve access to care, enhance patient outcomes, and promote health equity in underserved regions like rural Appalachia.
